# Automated verbal credibility assessment of intentions: The model statement technique and predictive modeling

**DOI:** 10.1002/acp.3407

**Published:** 2018-04-02

**Authors:** Bennett Kleinberg, Yaloe van der Toolen, Aldert Vrij, Arnoud Arntz, Bruno Verschuere

**Affiliations:** ^1^ Department of Psychology University of Amsterdam Amsterdam The Netherlands; ^2^ Department of Psychology University of Portsmouth Portsmouth UK

**Keywords:** credibility assessment, intentions, machine learning, model statement, verbal deception detection

## Abstract

Recently, verbal credibility assessment has been extended to the detection of deceptive intentions, the use of a model statement, and predictive modeling. The current investigation combines these 3 elements to detect deceptive intentions on a large scale. Participants read a model statement and wrote a truthful or deceptive statement about their planned weekend activities (Experiment 1). With the use of linguistic features for machine learning, more than 80% of the participants were classified correctly. Exploratory analyses suggested that liars included more person and location references than truth‐tellers. Experiment 2 examined whether these findings replicated on independent‐sample data. The classification accuracies remained well above chance level but dropped to 63%. Experiment 2 corroborated the finding that liars' statements are richer in location and person references than truth‐tellers' statements. Together, these findings suggest that liars may over‐prepare their statements. Predictive modeling shows promise as an automated veracity assessment approach but needs validation on independent data.

## 1 INTRODUCTION

On March 22, 2016, two suicide bombers detonated nail bombs at Brussels Airport in Zaventem, killing and seriously injuring many innocent civilians. In the aftermath of the terror attack, officials expressed concerns about the level of security, pointing to systematic security flaws and insufficient staff training at Brussels Airport (Bilefsky, 2016). This incident suggests that an additional screening of passengers *before* they arrive at the airport could be vital for the detection of aviation security threats. Although many existing methods aim at safeguarding aviation security, concerns have been voiced about the validity of these methods (Meijer, Verschuere, & Merckelbach, 2017; Ormerod & Dando, 2015). More research regarding the screening of airport passengers is needed to improve aviation safety. One possible line of inquiry is to explore whether one can differentiate between true and false intentions (Jupe, Leal, Vrij, & Nahari, 2017; Vrij, Granhag, Mann, & Leal, 2011).

### 1.1 Verbal deception detection

Among the more promising approaches to detect deception is examining the verbal content to discern truthful from deceptive statements (Bond & DePaulo, 2006; Oberlader et al., 2016). Verbal deception detection is rooted in the assumption that the verbal account of an event is informative about the veracity of that account. For example, genuine experiences are often reported differently than fabricated experiences, one of the core assumptions of reality monitoring (RM, Johnson & Raye, 1981). RM states that the differences are attributable to the process by which the memory of an event is constructed: Memories of truthfully experienced events have been obtained through perceptual processes, whereas fabricated memories were built through cognitive operations. Deception researchers adopted this idea and found promising results for verbal deception detection (Johnson, Bush, & Mitchell, 1998). Meta‐analytical findings support the notion that visual, auditory, and temporal details are useful in distinguishing truthful from deceptive accounts (Masip, Sporer, Garrido, & Herrero, 2005). Accuracy rates of classifying liars from truth‐tellers based on these variables are above chance level and range from 63% to 82% (Masip et al., 2005; Vrij, Fisher, & Blank, 2017; and see also Levine, Blair, & Carpenter, 2017; Vrij, Blank, & Fisher, 2018;).

### 1.2 Detecting deceptive intentions

For many years, deception research focused on people lying about their past actions (e.g., what someone was doing during the time of a crime). Since recently, attention is also paid to the detection of deceptive intentions (Mac Giolla, Granhag, & Liu‐Jönsson, 2013; Sooniste, Granhag, Knieps, & Vrij, 2013; Warmelink, Vrij, Mann, & Granhag, 2013). There are indications that the principles that apply to the detection of deception on past events also apply to deceptive intentions (Granhag & Mac Giolla, 2014). When truth‐tellers report a past event, they can rely on their memory, whereas liars cannot if they discuss an event they have never experienced. A similar logic may apply to lying about intentions. Plans for future actions that are not accompanied by an intention to execute result in a less detailed mental image of the event than plans that are accompanied by the enactment intentions (Granhag & Knieps, 2011; Szpunar, 2010). It is important to note, however, that past events are imagined in more detail than future events (D'Argembeau & Van der Linden, 2004; Gamboz et al., 2010). Cues for deception concerning intentions might, therefore, be less clear compared with those for past events.

To date, research into the verbal approach to the detection of deceptive intentions has examined different verbal cues with sometimes contradicting findings. In one study, passengers at international airports were instructed to lie or tell the truth about their forthcoming trip (Vrij et al., 2011). Those who lied about their journey provided statements that were less plausible and included more contradictions than truthful statements but did not differ in the amount of detail. Building on the notion that the expectedness of the questions asked might moderate the effectiveness of the verbal deception detection approach (Vrij & Granhag, 2012), another series of experiments asked participants expected and unexpected questions about a fabricated or truthful future event (Fenn, McGuire, Langben, & Blandón‐Gitlin, 2015; Warmelink et al., 2013; Warmelink, Vrij, Mann, Jundi, & Granhag, 2012). Although differences in the amount of detail emerged in some studies when unanticipated questions were asked (Sooniste et al., 2013; Warmelink et al., 2013), these effects were absent in other studies (Fenn et al., 2015; Kleinberg, Nahari, Arntz, & Verschuere, 2017). In yet another study, it was found that markers of good planning behavior (e.g., effective time allocation and *how* an action will be carried out) were more prevalent in truthful than in deceptive statements (Mac Giolla et al., 2013). Conversely, deceptive statements contained more justifications for the actions (i.e., *why* an action will be carried out). Furthermore, a recent study reported that deceptive intentions contained fewer verifiable details than truthful ones (Jupe et al., 2017). Taken together, the literature on the detection of deceptive intentions suggests that the verbal approach could be promising and that the richness of detail might be a useful cue to deception.

### 1.3 The model statement technique

A model statement is a detailed example of a verbal statement given by someone on a topic unrelated to the current research context, and providing such a statement may help to increase verbal differences between truth‐tellers and liars. By reading a detailed example before providing their account, interviewees are thought to learn the level of detail that is expected from their statement, which in turn makes them inclined to provide more detail. Providing more detailed information should be easier for truth‐tellers than for liars: The former could easily retrieve details from their memory of a specific event, whereas liars struggle to include sufficient detail to match the expectations raised by the model statement (Vrij, Fisher, & Blank, 2017; Vrij, Hope, & Fisher, 2014). Besides, liars will likely not provide more detailed information after reading a model statement because the provision of extra information could lead to cues that give away their lie (e.g., incriminating information, Nahari, Vrij, & Fisher, 2014) or expose the lack of contextual information in their account (Vrij, Fisher, & Blank, 2017).

There are mixed findings as to the usefulness of the model statement method so far. On the one hand, the provision of a model statement led to lengthier statements and better truth–lie discrimination (i.e., truthful statements were more plausible; see Leal, Vrij, Warmelink, Vernham, & Fisher, 2015). Another study found that the discrimination between truthful and deceptive insurance claims based on the number of verifiable details improved with a model statement (Harvey, Vrij, Leal, Lafferty, & Nahari, 2017). Moreover, a model statement benefited detection accuracy when details inferred from behavior scripts (e.g., “we went to the restaurant and ordered food and something to drink”) and complications were counted (Vrij, Leal, et al., 2017). These studies suggest that the model statement aids deception detection when the quality of information (e.g., plausibility, verifiability, and number of complications) is measured. On the other hand, several other studies have not found support for the beneficial role of a model statement when the quantity of details is examined. In Bogaard, Meijer, and Vrij (2014), a model statement led to lengthier statements but did not benefit the discrimination between truth‐tellers and liars with commonly used verbal content analysis tools measuring quantity of detail (e.g., RM). Likewise, there was no evidence to the beneficial effects of the provision of a model statement for the amount of “total details” (Ewens et al., 2016) nor for the statement quantity in children and adolescents (Brackmann, Otgaar, Roos af Hjelmsäter, & Sauerland, 2017). In sum, there are indications that a model statement may improve verbal deception detection when examining verbal aspects other than the quantity of details. Importantly, although some studies failed to find an effect of the model statement, no study indicated that a model statement impeded deception detection, and regarding quantity of details, several studies showed that it increased the information provided (e.g., Bogaard et al., 2014; Leal et al., 2015). The current study tests whether the model statement technique can facilitate the detection of truthful and deceptive intentions.

### 1.4 Large‐scale deception detection

In a setting such as prospective airport passenger screening, large‐scale deception detection may be only applicable when data can be collected and analyzed automatically (Kleinberg, Arntz, & Verschuere, in press). A key challenge for verbal deception detection is then the transition from manual, human coding of verbal content towards computer‐automated approaches. Although these two methodological lines have the same goal of identifying deceptive and truthful content, they both have different advantages and shortcomings (e.g., Hauch, Blandón‐Gitlin, Masip, & Sporer, 2015). First, the manual annotation of a text is limited in its large‐scale potential because it relies on instructed human coders. The efforts and time involved in the human coding approach make it virtually unfit for the assessment of vast numbers of statements in near real time (e.g., in airport settings). Computer‐automated approaches are less affected by this requirement and can be scaled up and allow for text analysis in real time (for a review, see Fitzpatrick, Bachenko, & Fornaciari, 2015). Second, inherent to the involvement of human assessors in manual coding is the lack of perfect reliability of the judgments made. Contrary to computer‐automated approaches, the agreement between multiple humans is never entirely perfect and therefore might pose a threat to the validity. Because we are particularly interested in potential large‐scale applications, we resort to computer‐automated methods as a primary analytical tool in the current study. Several methods have been proposed to integrate verbal deception theory and computer‐automated analysis.

#### 1.4.1 Linguistic Inquiry and Word Count

The Linguistic Inquiry and Word Count (LIWC) software (Pennebaker, Boyd, Jordan, & Blackburn, 2015) examines the proportion of words belonging to one of 92 categories. The attractiveness of the LIWC is that the categories are thought to represent psycholinguistic processes such as the emotional tone of a text (e.g., “lucky” and “melancholic”) or the number of cognitive processes in a text (e.g., “know” and “ought”). Each word category is composed of a comprehensive dictionary, and the analytical task consists of counting the number of words per category. Several studies have successfully used the LIWC to discriminate lies from truths (Bond & Lee, 2005; Kleinberg, Mozes, Arntz, & Verschuere, 2017; Mihalcea & Strapparava, 2009; Ott, Choi, Cardie, & Hancock, 2011; Pérez‐Rosas & Mihalcea, 2014).

#### 1.4.2 Named entity recognition

Recently, it has been proposed to use named entities in verbal deception detection (Kleinberg, Mozes, et al., 2017; Kleinberg, Nahari, & Verschuere, 2016). Named entity recognition (NER) is an information extraction method that identifies and classifies information from natural language into predefined categories (e.g., persons, dates, and times). Truthful statements are expected to contain more named entities than deceptive statements because truthful accounts (a) are typically richer in detail (Johnson et al., 1998; Masip et al., 2005), (b) contain more verifiable details (Nahari et al., 2014), and (c) are often more contextually embedded (Köhnken, 2004). The named entity‐based approach has been shown to be useful for the identification of deceptive and truthful hotel reviews (Kleinberg, Mozes, et al., 2017). These findings suggest that named entities might be a means to measure the liars' strategy of withholding potentially incriminating information (e.g., persons that could be consulted to verify an alibi), resulting in liars' mentioning fewer named entities.

### 1.5 The current study

We investigated whether it is possible to detect truthful and deceptive statements about planned activities in a computer‐automated verbal deception detection workflow (i.e., automated data collection and automated text analysis). Because the majority of verbal deception research has been conducted regarding past activities, we also included a comparison condition of participants who provided a truthful or deceptive statement about their recent activities (Experiment 1). To enhance verbal differences, we provided all participants with a model statement in Experiment 1 and experimentally investigated the provision of the model statement in Experiment 2.

In the first experiment, there were four conditions. In the two truthful conditions, participants were instructed to tell the truth about their (a) forthcoming or (b) past weekend. In the two deceptive conditions, participants were instructed to lie about an activity assigned to them (c) for the forthcoming or (d) about the past weekend. The main focus of this study was the automated detection of deception. All statements were therefore coded automatically using the LIWC and named entity approaches. Because human coding is the standard in the majority of psycholegal deception studies, we added manual annotations on a subset (40%) of the statements of Experiment 1.

We expected several main effects of veracity. On the basis of the theory of RM and the idea that richer mental images accompany genuinely planned activities, it was expected that truthful statements would be lengthier (dependent variable [DV]: no. of words), be richer in detail (DV: richness of detail measured via LIWC and human coding), contain more specific information (DV: named entities), and be more plausible (DV: human‐coded plausibility) than deceptive statements. We also expected that truthful statements would contain more references to *how* (DV: human‐coded how‐utterances) an activity was executed and fewer justifications of the actions (i.e., *why* they executed an activity, DV: human‐coded why‐utterances) than deceptive statements (Mac Giolla et al., 2013). Last, we expected that the difference between truthful and deceptive statements would be more pronounced for statements about the past than for statements about the future (interaction hypothesis). In the exploratory analysis, we looked at machine learning classification of truthful and deceptive statements and examined individual linguistic predictors.

### 1.6 Data availability statement

The confirmatory analyses for the two experiments were preregistered before data collection. The preregistrations, data, and supporting information are available at https://osf.io/wqc4p/. The source code to the experimental tasks is available at https://github.com/ben-aaron188/verbal_deception_past_future.

## 2 EXPERIMENT 1

The local institutional review board approved both experiments (#2016‐CP‐7306).

### 2.1 Method

#### 2.1.1 Participants

Data were collected through the online crowdsourcing website Prolific Academic (https://www.prolific.ac/) where we opened spots for 327 participants. Participation was open to all participants who were native English speakers and had not partaken in previous pilot studies. To ensure that participants had concrete weekend plans, we collected data just before a weekend (Thursday and Friday). All participants were reimbursed with GBP1.50 for this study. Due to simultaneous starting times, we collected data from 347 participants on which we applied four preregistered exclusion criteria: double IP addresses (*n* = 23), noncomplete data (*n* = 4), not following the instructions (*n* = 0), and failing the manipulation check (i.e., not recalling the instructions after writing the statement, *n* = 28; all participants were asked “How were you instructed to write your statement?” on a scale from 0 = answer truthfully to 100 = answer deceptively; we excluded those who indicated a score higher than 10 in the truthful condition, or a score lower than 90 in the deceptive condition).

The final sample of 292 participants was randomly allocated to one of the four experimental conditions: truthful statement about the past weekend (*n* = 73, 58.90% female, *M*_age_ = 33.92 years, *SD*_age_ = 11.43), deceptive statement about the past weekend (*n* = 60, 48.33% female, *M*_age_ = 35.55, *SD*_age_ = 11.54), truthful statement about the forthcoming weekend (*n* = 80, 60.00% female, *M*_age_ = 33.41, *SD*_age_ = 11.67), and deceptive statement about the forthcoming weekend (*n* = 79, 53.16% female, *M*_age_ = 33.71, *SD*_age_ = 10.05). There was no difference between the conditions in gender, *X* ^2^(3) = 2.42, *p* = .490, Cramer's *V* = 0.05, or age, *F*(1, 290) = 0.04, *p* = .837, *f* = 0.01.

#### 2.1.2 The model statement

We adhered to the suggested guidelines for formulating a model statement (Centre for Research and Evidence on Security Threats, 2016), with one exception. Given the online context of the current investigation, we did not provide an audiotaped version but rather presented the statement as text (as did Harvey et al., 2017). We followed the remaining suggestions and created a statement that (a) is unrelated to the research scenario (here: weekend plans), (b) describes an authentic experience, and (c) is not created on the spot during the interview.

The actual model statement was created by interviewing a friend of one of the authors via telephone about her first day at university. The interview was transcribed and translated into English from Dutch, resulting in a length of 527 words (Supporting Information S1). To ensure that the participants read the statement, they could only proceed to the next page after 1 min and were informed that they would be asked four multiple‐choice questions about the model statement (Supporting Information S2). If a participant failed to answer a question correctly, she or he was redirected to the model statement followed by four new multiple‐choice questions.

#### 2.1.3 Experimental manipulation

Participants were randomly allocated to one of two conditions of veracity (truthful vs. deceptive). Thus, participants gave either a deceptive or truthful statement on their planned or past activities. Liars were assigned an activity that they had to pretend to intend for the coming weekend (or have done on the past weekend). We allotted an activity to liars to avoid that they used one of their previously experienced weekend activities. To keep the selection of activities standardized, all participants had to choose from a drop‐down menu of 31 activities (e.g., attending a wedding; Supporting Information S3).

##### Past weekend plans

In the past weekend conditions, participants were asked to select at least one activity that they had carried out last weekend and at least three activities that they had not carried out last weekend. For those activities that they indicated to have carried out last weekend, they were asked to report how often they had done them before (on a slider from *never* to *very often*). Subsequently, they were asked the same question for the activities that they said they had not carried out last weekend. In the truthful condition, participants were instructed to provide a convincing account about one activity that was randomly chosen from their selected truthful activities. In the deceptive condition, participants were assigned one activity that they, in the previous step, indicated to not have carried out before. For instance, if a participant in the deceptive condition had indicated to have “visited the zoo” but did not “go to a birthday party,” the participant could be assigned to declare to have attended a birthday party. To provide a little more context, we added one extra detail to the selected activity in the deceptive condition. For example, if the determined activity was “throwing a party”, the assigned activity was “throwing a party with your friends at your favorite pub” (Supporting Information S4).

##### Future weekend plans

In the future weekend conditions, participants were asked to select at least one activity that they were planning to do on the upcoming weekend and at least three activities that they were not planning to do. For the planned activities, they were asked to indicate how often they had done them before, how certain they were about carrying out that activity, and how well they had planned that activity. For the activities that they indicated not to carry out, participants were asked how often they had carried them out before and how certain they were of not carrying them out. Equivalent to the truthful past weekend condition, those in the truthful forthcoming weekend condition were told one activity that they intended to do next weekend. In the deceptive forthcoming weekend condition, they were assigned the activity that had the lowest score on how often they had done it before and the highest score on how certain they were not to carry out that activity. Equivalent to the past weekend plans, we find a little more detail in the deceptive next weekend condition (e.g., “Going to a festival in a big city with a friend”).

#### 2.1.4 Procedure

Participants accessed the experimental task—advertised as “Lie detection study about your weekend plans”—via their Prolific account. The minimal requirement for doing this task was a Web browser. Upon starting the task, participants were informed about the study and gave their consent for participating. Next, they read general instructions about the purpose of the task that some participants are instructed to tell the truth about their last (or upcoming) weekend, and some are instructed to lie. On the next page, they gave information about their activities during last weekend or for the forthcoming weekend (see Section 2.1.3). Participants were then randomly allocated to an experimental condition and read instructions according to their veracity and time condition. In particular, participants were told that they were about to write a statement about one specific activity, which was indicated in bold letters alongside these instructions. Participants were then directed to the model statement. Once they proceeded through the model statement and the subsequent multiple‐choice test, participants received their statement instructions emphasizing that they should make their story “as detailed, plausible and convincing as possible.” In both veracity conditions, participants were reminded to write only about the given activity and that they could take the time to prepare their statement. Moreover, they were told that each account would be read by deception experts who would determine whether or not they believed the story. If they were believed, they would be rewarded with an additional GBP0.50. We paid the bonus to the participants with 20% highest overall proportion of named entities in their statement.

On the next screen, participants had to write their statement in a text box. They could only proceed to the next screen if their statement was at least 80 words long and if their statement was proper English. If these criteria were not met, they were reminded about the length and language of the required input via a pop‐up. We also disabled the copy‐and‐pasting functionality to prevent participants from reusing text.

After completing the statement, participants were asked three questions to be answered with a slider from 0 to 100.

1. “How were you instructed to write your statement?” (truthful–deceptive)
2. “How much of your statement is based on truthful elements?” (nothing–all of it)
3. “How motivated were you to write a convincing statement?” (not at all–absolutely)

Before exiting the experiment, all participants provided demographic information.

#### 2.1.5 Computer‐automated analysis

##### Linguistic Inquiry and Word Count

We used the LIWC to extract the proportions of words in each statement that belonged to those psycholinguistic LIWC categories that best represent the RM richness of detail. Specifically, we modeled the richness of detail as the sum of the LIWC categories *percept* (perceptual processes; including the subcategories *see*, *hear*, and *feel*; e.g., saw, touch, and heard), *space* (spatial references; e.g., down and in), and *time* (temporal references; e.g., until and end; Bond & Lee, 2005).

##### Named entity recognition

In contrast to lexicon approaches (e.g., LIWC), NER is rather flexible towards *unseen* words because it bases the information classification on probabilistic estimates derived from a supervised machine‐learning task (Nothman, Ringland, Radford, Murphy, & Curran, 2013). For example, it determines that “Harry Potter” is a person reference because it is more likely to be a person than, say, a date, location, or organization—without looking “Harry Potter” up in a database. By not relying on a lexicon, the NER approach can classify entities without having learned that information before. Here, we use the natural language processing library spaCy in the Python programming language (Version 1.3.0; Honnibal, 2016). We extract named entities of all the categories identified by spaCy: persons (e.g., “Chris”), nationalities or religious groups (e.g., “Chinese”), facilities (e.g., “Alum Chine”), organizations (e.g., “IKEA”), geopolitical entities (e.g., “South Korea”), locations (e.g., “Henver Road”), products (e.g., “VW”), events (e.g., “Birthday Party”), works of art (e.g., “Game of Thrones”), languages (e.g., “English”), dates (e.g., “2 nights”), times (e.g., “8 am tomorrow”), percentages (e.g., “50%”), money (e.g., “an additional $1.00”), quantities (e.g., “about 40 miles”), ordinals (e.g., “one”), and cardinals (e.g., “2nd”). Our outcome variable is the proportion of the occurrence of unique occurrences of named entities (i.e., each entity is counted only once) relative to the word count in each statement (Kleinberg, Mozes, et al., 2017).

#### 2.1.6 Manual coding of statements

A random subset of 147 statements (73 on past weekend plans and 74 on future weekend plans) was rated manually by two coders who were blind to the experimental condition and hypotheses. The coders were instructed to rate each statement as a whole on its plausibility, its richness of detail, the occurrence of *how*‐utterances and why‐utterances. 1Plausibility: “Could this incident have happened as described? Could this be an honest description of someone's weekend activities?” (Leal et al., 2015). Richness of detail: “The inclusion of specific descriptions of place, time, persons, objects and events in the statement” (Vrij, 2015). The occurrence of *how*‐utterances: “Concrete descriptions of activities. This can include, but is not limited to, sentences that included phrases such as ‘we planned to…’, ‘we were going to…’, ‘we intended to…’” (Mac Giolla et al., 2013). Why‐utterances: “There are two types of answers to ‘why’. First, wider motivations/reasons why someone planned an activity. Second, motivations/reasons for doing something in a certain way” (Mac Giolla et al., 2013). Each variable was scored on a Likert scale from 1 (*very low/few*) to 7 (*very high/many*). Although recent findings suggest that counting details is more reliable than scale judgments (Nahari, 2016), we decided to follow the procedure of previous intentions studies (Sooniste, Granhag, Strömwall, & Vrij, 2015).

Both coders received a training session in which statements were rated and discussed with an instructor. Further, 40% of the statements (*n* = 58) were rated by both coders, and the remaining 60% (*n* = 88) were randomly split between the two coders. The intraclass correlation coefficients were .11 for plausibility (*ns*), .90 for richness of detail (*p* < .001), .60 for how‐utterances (*p* < .001), and .67 for why‐utterances (*p* < .001). Because of the very low reliability of plausibility, we decided not to analyze plausibility judgments.

## 3 RESULTS

### 3.1 Analytical plan

We conducted separate 2 (veracity: truthful vs. deceptive) by 2 (time: past vs. future) between‐subjects ANOVAs with preregistered Bonferroni significance level correction on each of the DVs. For seven key DVs in the main, preregistered, analysis, we adhered to an alpha significance level of .05/7 = .007. The effect size Cohen's *f* indicates the magnitude of effects, with *f* = 0.10, *f* = 0.25, and *f* = 0.40 for small, moderate, and large effects, respectively (Cohen, 1988).

To compare the diagnostic efficiency of the DVs, we conducted receiver operating characteristics analyses. We compare the areas under the curve (AUCs) using Venkatraman's (2000) AUC comparison test. In the exploratory analyses, we used a supervised machine learning classification task to predict the veracity of statements. All statistical analyses were conducted with R (R Core Team, 2016). For AUC analysis, we used the *pROC* R package (Robin et al., 2011). The machine learning analyses were conducted with the *caret* package (Kuhn, 2017).

### 3.2 Confirmatory analysis

Table 1 summarizes the results for the confirmatory analyses, expecting main effects of veracity. There was no significant interaction effect between veracity and time for any of the DVs. For the number of words and how‐utterances, a significant main effect of time revealed that the statements were lengthier and contained more how‐utterances when they were about past weekend activities than when they were about forthcoming weekend plans. Only for one of the four human‐coded DVs was the hypothesis supported: truth‐tellers included more how‐utterances in their statement than liars.

**Table 1:** Summary table with confirmatory analyses for Experiment 1 (*M*, *SD*, Cohen's *d*)

Dependent variable	Past		Future		Main effect veracity	Main effect time	Veracity * Time Interaction	Hyp.	Expected truth–lie difference supported?
	Truthful	Deceptive	Truthful	Deceptive					
Number of words	261.68 (141.65)	284.12 (172.92)	233.72 (139.92)	210.38 (114.88)	0.00 (*p* = .978)	0.18* (*p* = .003)	0.08 (*p* = .172)	T > D	No
Richness of detail (LIWC)	19.26 (4.39)	19.20 (2.88)	17.83 (4.49)	18.04 (4.39)	0.01 (*p* = .880)	0.16 (*p* = .009)	0.02 (*p* = .777)	T > D	No
% of named entities	3.35 (2.18)	4.16 (1.60)	3.90 (1.89)	3.85 (2.06)	0.10 (*p* = .101)	0.03 (*p* = .605)	0.11 (*p* = .065)	T > D	No
Richness of detail (human coded)	4.22 (1.64)	4.97 (1.24)	4.43 (1.34)	4.14 (1.45)	0.08 (*p* = .336)	0.11 (*p* = .193)	0.18 (*p* = .031)	T > D	No
How‐utterances (human coded)	5.16 (1.24)	4.63 (0.80)	4.60 (1.04)	4.00 (1.13)	0.26* (*p* = .002)	0.28* (*p* = .001)	0.02 (*p* = .847)	T > D	Yes
Why‐utterances (human coded)	3.23 (1.26)	3.20 (1.40)	3.24 (1.06)	3.25 (1.44)	0.00 (*p* = .974)	0.01 (*p* = .907)	0.01 (*p* = .920)	D > T	No

* *p* < .007.

### 3.3 Exploratory analyses

#### 3.3.1 Machine learning classification: Experiment 1

To predict the veracity of a statement, we used supervised machine learning classification, which, contrary to classical statistical testing, learns from the data to predict an outcome (for an overview, see Yarkoni & Westfall, 2017). More specifically, in a supervised machine learning task, a classifier algorithm is trained on a subset of the data to predict an outcome class (here: truthful vs. deceptive). To build a classifier algorithm, one selects features (i.e., predictor variables) based on which the relationship to the outcome class is learned. To avoid overfitting, we split the data into a training set (80% of the data) and a holdout test set (20%). During the training phase, we applied a fivefold cross‐validation with 10 repetitions (e.g., Ott et al., 2011). The cross‐validation procedure ensures that each observation in the training data has been used for building and validating the final predictive model. Once the final model was determined, we assessed the performance on the holdout test set, which was not used in the training phase. This procedure is used as a safeguard to ensure the validity of the final model.

We used the commonly applied linear support vector machine (SVM) as a classifier (Mihalcea & Strapparava, 2009; Ott et al., 2011). Linear SVMs create an *n*‐dimensional space, where *n* equals the number of features and calculates a linear kernel function that splits the data into two classes (here: truthful and deceptive). The aim is to derive a hyperplane that splits the data in a way that the distance between the hyperplane and the two classes in the *n*‐dimensional space is maximized (Murphy, 2012).

As feature sets, we used (a) all LIWC variables (92 features) and (b) a subset intended to model psychological processes (40 features, e.g., cognitive processes, negative thinking, perceptual processes, see Supporting Information S6). Table 2 shows the performance metrics for both past and forthcoming weekend plans.

**Table 2:** Accuracies of the supervised machine learning task (linear support vector machine) for two different LIWC feature sets

Feature set	Data	Accuracy [95% CI]	Sens.	Spec.	AUC (95% CI)
Complete LIWC	Past weekend plans	69.23 [48.21, 85.67]	71.43	66.67	0.70 [0.48, 0.91]
	Forthcoming weekend plans	80.65 [62.53, 92.55]^a^	62.50	100.00	0.75 [0.56, 0.94]
Psychological processes	Past weekend plans	61.54 [40.57, 79.99]	78.87	41.67	0.77 [0.58, 0.96]
	Forthcoming weekend plans	74.19 [55.39, 88.14]^a^	62.50	86.67	0.78 [0.62, 0.94]

*Note*. LIWC = Linguistic Inquiry and Word Count; Sens. = sensitivity; Spec. = specificity.

a Significantly better than the chance level.

The findings suggest the predictive models built on all LIWC variables and the “psychological processes” subset outperform chance classification for prospective weekend plans but not for past weekend plans.

#### 3.3.2 Other LIWC variables and individual named entities

We explored whether truth–lie differences emerged on individual LIWC or named entity categories. This also enabled us to understand the verbal differences within the composite score of “richness of detail.” Table 3 displays the means and effect size of the veracity main effect for the three LIWC subcategories that formed the LIWC richness of detail (i.e., percept, space, and time) and other individual LIWC and named entity categories that were significant veracity predictors in another study with the same approach (Kleinberg, Mozes, et al., 2017). The findings suggest that although the categories percept (*f* = −0.12), space (*f* = −0.17), and time (*f* = 0.23) were significant in differentiating deceptive from truthful statements, they did exhibit their effect in different directions. Only the temporal information category (“time”) was, as could be expected from RM, higher for truthful than for deceptive statements. The spatial information (“space”) and perceptual processes (“percept”) were higher in deceptive than in truthful texts. These discrepant findings might explain why the composite index of the LIWC richness of detail did not indicate a significant difference.

**Table 3:** Means (*SD*s, Cohen's *d*) for the dependent variables used in the exploratory analyses per time and veracity

Dependent variable	Main effect veracity	Past weekend plans			Future weekend plans		
		Truthful	Deceptive	Main effect veracity	Truthful	Deceptive	Main effect veracity
Richness in detail: percept	−0.12*	1.93 (1.37)	2.10 (1.26)	−0.06	1.52 (1.53)	1.99 (1.30)	−0.17*
Richness in detail: time	0.23*	8.96 (3.05)	8.09 (2.01)	0.12	8.75 (3.41)	7.16 (2.36)	0.27*
Richness in detail: space	−0.17*	8.38 (2.67)	9.02 (2.56)	−0.17	7.56 (2.96)	8.90 (3.43)	−0.21*
Function words (function)	−0.16*	53.49 (4.08)	55.35 (3.12)	−0.25*	55.89 (3.99)	56.46 (3.90)	−0.07
Personal pronouns (ppron)	−0.09	9.67 (2.70)	10.79 (2.30)	−0.22*	10.69 (2.56)	10.53 (2.53)	0.03
First person singular (i)	0.24**	6.53 (2.70)	5.15 (2.69)	0.26*	6.80 (3.54)	5.40 (2.55)	0.23*
Numbers (number)	0.12*	1.86 (1.42)	1.57 (1.03)	0.12	1.70 (1.47)	1.41 (1.04)	0.11
Persons	−0.32*	0.29 (0.49)	0.76 (0.70)	−0.39*	0.34 (0.63)	0.73 (0.77)	−0.27*
Geopolitical entities	−0.25*	0.17 (0.45)	0.48 (0.59)	−0.30*	0.27 (0.51)	0.51 (0.66)	−0.21*
Dates	0.13*	1.11 (0.77)	1.06 (0.62)	0.03	1.56 (0.98)	1.17 (0.89)	0.21*
Time	0.12*	0.54 (0.68)	0.56 (0.52)	−0.02	0.53 (0.59)	0.29 (0.42)	0.24*
Ordinal	0.17**	0.24 (0.39)	0.09 (0.20)	0.25*	0.13 (0.28)	0.09 (0.22)	0.08

*Note*. Negative effect sizes imply higher values in deceptive than in truthful statements.

* *p* < .05.

** *p* < .01.

Table 3 further shows that persons (*f* = −0.32) and geopolitical entities (*f* = −0.25) were the best discriminators but were both more frequent in deceptive statements than in truthful statements. Further, the occurrence of date (*f* = 0.13) and time (*f* = 0.12) references as well as of ordinals (*f* = 0.17) was significantly higher in deceptive than in truthful statements. Because there were no hypotheses about these specific findings, a replication experiment is needed to identify the robustness of these (unexpected) findings.

### 3.4 Discussion: Experiment 1

The confirmatory analysis of the first experiment showed that deceptive statements did not differ from truthful statements in length, the richness of detail, named entities, and why‐utterances. We found support only for the hypothesis that truthful statements would contain more how‐utterances than deceptive ones. The exploratory predictive analysis yielded promising results for machine learning classification tasks. Deceptive and truthful plans for the forthcoming weekend were identified with an accuracy above chance (80.64% and 74.19% for all LIWC variables and psychological processes, respectively). Exploratory analysis also suggested that liars included more references to persons and places than truth‐tellers. However, this result may be due to a confound: Liars received slightly more specific instructions for their activities (e.g., “Going on a holiday to Spain with a friend”) than truth‐tellers (e.g., “Going on a holiday”). As such, the inclusion of person and place references may have been a function of the instructions rather than the veracity. To further investigate these seemingly contradictory findings and to assess the replicability of the predictive modeling results, we ran a second experiment with preregistered hypotheses. The second experiment also allowed us to isolate the effect of the model statement technique. Because we were mainly interested in the emerging area of detecting deceptive intentions, in the second experiment, we collected data on future weekend plans only and manipulated the veracity of the statements as well as the provision of a model statement. We further adjusted the instructions so that both liars and truth‐tellers were given identical instructions when writing their statement.

Moreover, recently, there has been a criticism that a cross‐validation procedure of prediction models of any kind is lacking in the psycholegal verbal deception research literature and has likely resulted in overestimates of the reported accuracies (Levine et al., 2017). We decided to extend the cross‐validation from Experiment 1 by validating the models from Experiment 1 with data from a new sample in Experiment 2.

## 4 EXPERIMENT 2

Experiment 2 served four purposes. First, we wanted to replicate the findings obtained in the machine learning analysis on data from an independent sample. Second, the potential confound of different instructions to liars and truth‐tellers was corrected. Third, we wanted to test whether the significant (and unexpected) differences found in the exploratory analysis of Experiment 1 for individual LIWC and named entity categories could be replicated. Fourth, we manipulated the provision of the model statement to examine whether a model statement is beneficial to the detection of deceptive and truthful forthcoming weekend plans. Because the primary interest of this investigation is the detection of deceptive intentions, all participants were asked to write about their plans for the coming weekend. Furthermore, because the analytical focus of this investigation is on potentially scalable methods, we used only automated analyses in Experiment 2. On the basis of the findings from Experiment 1 and from studies that show the beneficial effect of the model statement technique (Harvey et al., 2017; Leal et al., 2015), we preregistered the following hypotheses:

- Deceptive statements will contain more (computer‐scored) person, location, temporal, spatial, date, and time references than deceptive statements.
- The machine learning classification accuracy of truthful and deceptive statements is above chance level. The classifier trained on the data of Experiment 1 performs with above chance level accuracy on the data of Experiment 2.
- The differences in linguistic and verbal content variables between truthful and deceptive statements are larger when a model statement is provided than when it is not, resulting in higher classification accuracy.

### 4.1 Method

#### 4.1.1 Participants

The data collection procedure was identical to that of Experiment 1. We aimed to replicate the effects found in the first experiment and adhered to the same sample size including a buffer for potential data loss, resulting in 100 participants required per condition. Due to simultaneous starting times, we collected data of 413 participants and, as per the preregistered exclusion criteria, excluded those who could not recall whether they were instructed to write a truthful or deceptive statement after writing the statement (*n* = 28, final sample = 385). 2The IP exclusion was obsolete and not preregistered because Prolific Academic has several control mechanisms built in to prevent multiple participations per participant. The remaining 385 participants were allocated blockwise into four experimental conditions: a truthful condition with a model statement (*n* = 90, 66.67% female, *M*_age_ = 32.56 years, *SD*_age_ = 9.23), a deceptive condition with a model statement (*n* = 97, 70.10% female, *M*_age_ = 32.39, *SD*_age_ = 10.42), a truthful condition without a model statement (*n* = 101, 73.27% female, *M*_age_ = 32.00, *SD*_age_ = 9.36), and a deceptive condition without a model statement (*n* = 97, 69.07% female, *M*_age_ = 33.55, *SD*_age_ = 11.06). There was no difference between the conditions in gender, *X* ^2^(3) = 1.02, *p* = .795, Cramer's *V* = 0.03, or age, *F*(1, 383) = 0.24, *p* = .626, *f* = 0.03.

#### 4.1.2 Changes compared with Experiment 1

Those who read the model statement followed the same procedure as those in Experiment 1. Participants who did not read a model statement were directed to the input field immediately after they received their veracity instructions (including the prompt to be as detailed, plausible, and convincing as possible). This procedure was based on related previous studies (Bogaard et al., 2014; Leal et al., 2015). The instructions provided to deceptive participants were changed to be identical to those given to truth‐tellers; that is, all participants received the nonspecific instructions (e.g., “throwing a party”).

### 4.2 Results

#### 4.2.1 Confirmatory analyses

Table 4 shows that the findings of Experiment 1 were supported for person references and location references, which were both more prevalent in deceptive than in truthful statements. There were no veracity‐by‐model statement interaction effects. For person references (with > without model statement) as well as for temporal information (without > with a model statement) and date references (without > with a model statement), there was a significant main effect of the provision of the model statement, albeit only for person references in the expected direction. 3For an exploration of automating how‐ and why‐utterances, see Supporting Information S7.


**Table 4:** Summary table with the confirmatory analyses for Experiment 2 (*M*, *SD*, Cohen's *d*)

Dependent variable	Without model statement		With model statement		Main effect veracity	Main effect model statement	Veracity * Model Statement	Hyp.	Expected truth–lie difference supported?
	Truthful	Deceptive	Truthful	Deceptive					
Person references (NER)	16.58 (51.59)	23.59 (51.22)	23.68 (48.18)	40.23 (54.53)	−0.11* (*p* = .026)	0.12* (*p* = .025)	0.04 (*p* = .365)	D > T	Yes
Location references (NER)	18.91 (49.71)	29.67 (57.63)	24.55 (57.81)	42.82 (68.69)	−0.12* (*p* = .016)	0.08 (*p* = .118)	0.03 (*p* = .532)	D > T	Yes
Temporal information (LIWC)	9.10 (3.79)	9.06 (3.92)	7.91 (2.69)	8.01 (3.07)	0.01 (*p* = .941)	0.16* (*p* = .002)	0.01 (*p* = .849)	T > D	No
Spatial information (LIWC)	7.56 (3.58)	7.81 (2.93)	7.86 (3.11)	7.88 (2.96)	0.02 (*p* = .680)	0.03 (*p* = .562)	0.02 (*p* = .727)	D > T	No
Date references (NER)	170.54 (124.20)	175.76 (132.47)	133.89 (93.32)	139.11 (97.44)	0.02 (*p* = .652)	0.16* (*p* = .002)	0.00 (*p* = .999)	T > D	No
Time references (NER)	52.79 (87.51)	36.91 (61.22)	45.60 (55.57)	48.95 (55.99)	0.05 (*p* = .358)	0.02 (*p* = .722)	0.07 (*p* = .159)	T > D	No
Number of words	121.83 (57.37)	118.55 (48.54)	202.88 (107.36)	188.43 (93.47)	0.06 (*p* = .276)	0.48*** (*p* < .001)	0.04 (*p* = .493)	—	—

*Note*. Negative effect sizes imply higher values in deceptive than in truthful statements. LIWC = Linguistic Inquiry and Word Count; NER, named entity recognition.

* *p* < .05.

** *p* < .01.

*** *p* < .001.

##### Machine learning classification: Experiment 2

We predicted that the overall classification accuracy with a machine learning approach would be significantly better than chance level. Specifically, we predicted that when with all LIWC categories, the resulting classification accuracy was better than the chance level (here: 50.39% due to a slight condition imbalance). The machine learning classification resulted in an accuracy of 67.11% (95% CI [55.37%, 77.46%]) with AUC = 0.69 (95% CI [0.57, 0.82]; sensitivity = 68.42%, specificity = 65.79%). An exact binomial test revealed that accuracy was significantly higher than chance (*p* = .002).

We also predicted that the classification accuracy would be higher when a model statement was provided than when participants did not read a model statement. When a model statement was provided, we found an accuracy of 62.16% (44.76–77.54%) with AUC = 0.66 (95% CI [0.48, 0.84]; sensitivity = 38.89%, specificity = 84.21%), which was not better than chance (*p* = .125). Without a model statement, the accuracy was 56.41% (39.62–72.10%) with AUC = 0.63 (95% CI [0.45, 0.82]; sensitivity = 65.00%, specificity = 47.37%, *ns*, *p* = .316). 4The results show that the accuracy on the whole dataset is better than on both separate subsets (model statement and no model statement). This is likely due to the sample size used to train the classification models, whereby larger samples contain more information to be used in the predictive model. We expected that the diagnostic efficiency of the classifier for participants with the model statement would be significantly better than for the participants who did not read the model statement. There was no difference between the two classifiers, Venkatraman's AUC comparison test (*E* = 0.04, 2,000 bootstraps, *p* = .868). Note also that both classifiers' accuracy did not outperform chance level.

##### Cross‐experiment machine learning classification

To assess the classification accuracy of machine learning classifiers on independent data, we used the exact SVM classifier with the full LIWC feature set of the intentions data from Experiment 1 and tested its performance on the data from Experiment 2. That is, rather than evaluating the performance on holdout data from the same data collection, we test it on truly independent data from a different sample. This analysis resulted in an accuracy of 61.30% (56.23–66.19%) with an AUC of 0.64 (95% CI [0.59, 0.70]; sensitivity = 68.59; specificity = 54.12, *p* < .001). Moreover, when we tested the classifier on the data of participants who read the model statement (i.e., identical to Experiment 1), the accuracy was 63.10% (55.75–70.03%; AUC = 0.64, 95% CI [0.57, 0.72]; sensitivity = 66.67; specificity = 59.79, *p* = .001).

#### 4.2.2 Exploratory analysis

For comparison purposes, we also explored the length of statement (Table 4) as a function of veracity and the model statement. As in previous research, statements were lengthier when participants read the model statement (*M* = 188.35, *SD* = 97.24) than when they did not (*M* = 115.94, *SD* = 51.67). The findings are in line with previous research showing that a model statement increased information provided by the participants (Bogaard et al., 2014; Leal et al., 2015).

## 5 GENERAL DISCUSSION

This study examined whether the statements written about someone's weekend plans can reveal his or her veracity. In two experiments, participants wrote either a deceptive or truthful statement about their planned activities on the forthcoming weekend. In the first experiment, all participants read a detailed model statement and were asked to lie or tell the truth about their weekend plans. The theory of verbal deception detection predicts that truthfully intended activities can be recalled in more detail and contain more planning markers and fewer justifications for the intended actions than deceptive intentions. Because the primary aim of this study was to test the detectability of deceptive intentions in a potentially large‐scale setting, we collected data through an online interface and focused on computer‐automated analysis.

### 5.1 Predicting the veracity of statement

From an applied perspective, such as prospective passenger screening, the prediction accuracy of a model might be more important than the explanatory aspects underlying it. With the use of machine learning, deceptive and truthful statements were classified well above chance with relatively high accuracies of 74.19% and 80.65%, respectively. To assess the “true” performance of a predictive model, it is important to test it on newly collected data. In fact, most machine learning approaches to verbal deception detection are not evaluated on data from a new sample (Fitzpatrick et al., 2015), and most of the reported accuracy rates in the psycholegal literature were obtained without any cross‐validation (see the critique by Levine et al., 2017). We, therefore, examined the robustness of these accuracy rates with cross‐validation within the sample as well as on a new sample in the second experiment. The current investigation is, to the best of our knowledge, the only one that tested a classifier's accuracy on fresh, independent data from a new sample. The results are promising in that they withstood the cross‐experiment test, but they also highlight the drop of the accuracy when classifiers were applied to out‐of‐sample data. The accuracy rates will per definition be higher if the classifier is trained and tested on the same data, compared with a proper validation on a new sample (Yarkoni & Westfall, 2017). Although data from the first experiment suggest accuracies of up to 80%, the independent‐sample validation indicated that the true boundaries might be closer to 63% (similar accuracies using automated analysis were achieved by Pérez‐Rosas & Mihalcea, 2014). We strongly recommend that future research that makes claims about prediction incorporate a cross‐validation (e.g., train–test split or leave‐one‐out cross‐validation) and proper, actual validation on a new sample to avoid the reporting of overestimated accuracies. In the current study, without proper validation on a new sample, the reported accuracies would have been falsely exaggerated by more than 25%.

### 5.2 Do liars over‐prepare their statement?

As expected, past weekend activities were, in general, lengthier and contained more planning markers than statements about the forthcoming weekend. This effect is in line with other studies showing that experienced events can be recalled in more depth than not yet experienced events (D'Argembeau & Van der Linden, 2004). We found support for the hypothesis that truthful statements contain more indicators of careful planning (i.e., how‐utterances) than false ones, which might be attributable to the motivation of actually executing the plan, whereas fabricated intentions do not evoke such a motivation (Mac Giolla et al., 2013). Critically, however, there were no differences in the length, the richness of detail, or justifications between truthful and deceptive accounts.

Although no differences emerged in the computer‐automated extraction of the richness of detail (LIWC) and the specificity of information (named entities), exploratory analyses hinted at unexpected underlying dynamics of deceptive and truthful accounts: In line with the theory, truthful statements about intentions contained more temporal information, more time, and more date references than deceptive ones. However, contrary to the expectation, deceptive statements contained more person entities, more place entities, and more spatial information. Theoretical lines would predict that these kinds of aspects are rather unlikely for liars because they would offer potentially checkable details (e.g., a person to consult or a CCTV camera at a specific place to examine). To assess whether these findings replicate, we preregistered a second experiment where we hypothesized the observed, unexpected dynamics. Moreover, the second experiment excluded a potential confound in the instructions (i.e., adding a person or location reference to the liars' instructions) and experimentally manipulated the presence of the model statement.

Did the unexpected findings for location and person entities replicate? The effect sizes of the location entities (Experiment 1: *f* = −0.21; Experiment 2: *f* = −0.12) and person entities (Experiment 1: *f* = −0.27; Experiment 2: *f* = −0.11) were smaller in the second experiment. One reason for the decrease in the magnitude of the truth–lie differences could be that Experiment 2 did not contain the confounding, overly specific instructions of Experiment 1. If this were the case, the corroboration of these counterintuitive findings is even more interesting because it suggests that even without any hint at persons or locations, liars tend to include significantly more of these entities. Interestingly, comparable findings were reported in a study about a forthcoming trip (Warmelink et al., 2012). When asked about their intention (“What is the main purpose of your trip?”), liars reported significantly more detail than truth‐tellers, and vice versa for less expected questions (“How are you going to travel to your destination?”). There are two potential explanations for the current findings. First, liars might have simply chosen to bluff. Possibly, this strategy is specific for the online data collection context applied here, with liars being aware that the information about a future event would be difficult to check. Second, liars might have prepared more for the statement and might have been preoccupied with a detailed, convincing yet false account. Truth‐tellers, in contrast, could have relied on the idea that their truthfulness “shines through” (“the illusion of transparency,” Vrij, Granhag, & Porter, 2010, p. 109) without the need to prepare extensively. Tentative support in that direction stems from post hoc analysis on the time needed to write the statement (seconds per word): In the second experiment, liars took longer (*M* = 3.17 sec./word, *SD* = 2.94) than truth‐tellers (*M* = 2.52, *SD* = 1.31, *f* = 0.14, *p* < .001). This trend was not significant for Experiment 1 (*M*_t_ = 2.51, *SD*_t_ = 1.87, *M*_d_ = 2.64, *SD*_d_ = 1.22, *f* = 0.04, *ns*).

Liars might find it difficult to imagine what a truthful statement about an intended action might look like so that they include unrealistically many specific pieces of information out of precaution to sound believable. If this were the case, the naiveté of liars might possibly work in their disadvantage and give away their deceit. It would be interesting for future research to use questions that asks about things that truth‐tellers typically do *not* have an answer for.

### 5.3 The model statement technique

We did not find support for the hypothesis that providing a model statement benefits deception detection. Unexpectedly, participants who read a model statement provided fewer date entities and temporal information but more person entities than those who did read a model statement. These latter findings would need corroboration. The absence of a beneficial effect of the model statement was also reported elsewhere (Bogaard et al., 2014; Brackmann et al., 2017; Ewens et al., 2016; Harvey et al., 2017; Leal et al., 2015; Vrij, Leal, et al., 2017). We see two possible explanations. First, hidden moderators might determine the role of the model statement. Looking at the verbal cues—especially details, at a more granular level (e.g., qualifying details into verifiable details, script behavior details, and complications)—could be an important aspect for further research (the data of the two experiments are openly available). Second, the null findings might be due to boundary conditions of the model statement technique. We provided participants with a model statement about a *past* event (i.e., first day at uni). Future research could assess whether an alignment of the temporal focus of the model statement and the participants' action (i.e., past or future action) is necessary. Furthermore, the length requirement that we imposed on all statements (minimum of 80 words) could have played a role. Although intended as a safeguard to elicit sufficient information in the online context, it is possible that this resulted in unnatural content and blurred potential truth–lie differences.

#### 5.3.1 Manual versus automated text analysis

Concerning the large‐scale focus in this study, two aspects merit attention.

1. Although the computer‐automated analysis was applied successfully above chance level in the current study, the value of manual human scoring cannot (yet) be dismissed. Semantic, linguistic concepts such as plausibility are not yet easily automatable. Likewise, promising approaches such as the verifiability approach (i.e., looking at verifiable details, Nahari et al., 2014) are currently limited to manual annotation, which limits their large‐scale potential. The technical question of human versus automated coding performance might best be answered in direct comparisons and rigorous empirical testing. Such a comparison should test which technique yields the best accuracies and, most importantly, produces replicable and generalizable results. Because the aim of the current paper was to predict the veracity rather than illuminate the theoretical underpinnings of it, we focused more on the machine learning part rather than the individual cues underpinning it. We do acknowledge that the theory matters and should, in fact, be incorporated into predictive models to make use of the best of both worlds. In the future, hybrid approaches (e.g., Kleinberg, Mozes, et al., 2017) might help bridge the gap between theory and methods and human and automated analyses: Human annotations of the verifiability, for example, could be used as outcome variables for a predictive linguistic model. Ideally, this could result in a real‐time and valid proxy for otherwise manually coded constructs.
2. The current study relied on a passive collection of data. Alternatively, future approaches could explore how dynamic conversational environments (e.g., online chat) facilitate deception detection. Such a line of inquiry might also help to shorten the participation duration which is essential for applied purposes and would allow for the targeted elicitation of needed information (e.g., those pieces that could be verified).

## 6 CONCLUSION

Verbal deception detection is a promising yet complex path for the detection of deceptive intentions—both from an academic and from an applied perspective. In two experiments, we found evidence that liars mentioned more person and location references than truth‐tellers, which may be exploited for the detection of their false accounts. Predictive modeling with psycholinguistic features yielded promising results above chance level. At the same time, independent validation showed that within‐sample cross‐validation might still overestimate classification accuracies. The current findings provide novel insights into liars' strategies, highlight the promise of machine learning for deception detection, and emphasize the need for proper validation of predictive deception detection analysis.

## References

[acp3407-bib-0001] Bilefsky, D. (2016, March 31). Brussels attacks renew criticism of security at Europe's airports. *The New York Times* Retrieved from https://www.nytimes.com/2016/04/01/world/europe/brussels-attacks-airport-security.html

[acp3407-bib-0002] Bogaard, G. , Meijer, E. H. , & Vrij, A. (2014). Using an example statement increases information but does not increase accuracy of CBCA, RM, and SCAN: Using an example statement with truth tellers and liars. Journal of Investigative Psychology and Offender Profiling, 11(2), 151–163. https://doi.org/10.1002/jip.1409

[acp3407-bib-0003] Bond, C. F. , & DePaulo, B. M. (2006). Accuracy of deception judgments. Personality and Social Psychology Review, 10(3), 214–234. https://doi.org/10.1207/s15327957pspr1003_2 16859438 10.1207/s15327957pspr1003_2

[acp3407-bib-0004] Bond, G. D. , & Lee, A. Y. (2005). Language of lies in prison: Linguistic classification of prisoners' truthful and deceptive natural language. Applied Cognitive Psychology, 19(3), 313–329. https://doi.org/10.1002/acp.1087

[acp3407-bib-0005] Brackmann, N. , Otgaar, H. , Roos af Hjelmsäter, E. , & Sauerland, M. (2017). Testing a new approach to improve recall in different ages: Providing witnesses with a model statement. Translational Issues in Psychological Science, 3(2), 131–142. https://doi.org/10.1037/tps0000116

[acp3407-bib-0006] Centre for Research and Evidence on Security Threats . (2016). CREST guide: The model statement technique. Retrieved from https://crestresearch.ac.uk/resources/model-statement-technique/

[acp3407-bib-0007] Cohen, J. (1988). Statistical power analysis for the behavioral sciences. New York, NY: Academic Press.

[acp3407-bib-0008] D'Argembeau, A. , & Van der Linden, M. (2004). Phenomenal characteristics associated with projecting oneself back into the past and forward into the future: Influence of valence and temporal distance. Consciousness and Cognition, 13(4), 844–858. 15522635 10.1016/j.concog.2004.07.007

[acp3407-bib-0009] Ewens, S. , Vrij, A. , Leal, S. , Mann, S. , Jo, E. , Shaboltas, A. , … Houston, K. (2016). Using the model statement to elicit information and cues to deceit from native speakers, non‐native speakers and those talking through an interpreter: Using the MS to elicit information. Applied Cognitive Psychology, 30(6), 854–862. https://doi.org/10.1002/acp.3270

[acp3407-bib-0010] Fenn, E. , McGuire, M. , Langben, S. , & Blandón‐Gitlin, I. (2015). A reverse order interview does not aid deception detection regarding intentions. Frontiers in Psychology, 6 Retrieved from https://www.ncbi.nlm.nih.gov/pmc/articles/PMC4553365/ 10.3389/fpsyg.2015.01298 PMC4553365 26379610

[acp3407-bib-0011] Fitzpatrick, E. , Bachenko, J. , & Fornaciari, T. (2015). Automatic detection of verbal deception (Vol. 8). Morgan & Claypool Retrieved from http://www.morganclaypool.com/doi/abs/10.2200/S00656ED1V01Y201507HLT029

[acp3407-bib-0012] Gamboz, N. , De Vito, S. , Brandimonte, M. A. , Pappalardo, S. , Galeone, F. , Iavarone, A. , & Della Sala, S. (2010). Episodic future thinking in amnesic mild cognitive impairment. Neuropsychologia, 48(7), 2091–2097. 20381507 10.1016/j.neuropsychologia.2010.03.030

[acp3407-bib-0013] Granhag, P. A. , & Knieps, M. (2011). Episodic future thought: Illuminating the trademarks of forming true and false intentions. Applied Cognitive Psychology, 25(2), 274–280. https://doi.org/10.1002/acp.1674

[acp3407-bib-0014] Granhag, P. A. , & Mac Giolla, E. (2014). Preventing future crimes: Identifying markers of true and false intent. European Psychologist, 19(3), 195–206. https://doi.org/10.1027/1016-9040/a000202

[acp3407-bib-0015] Harvey, A. C. , Vrij, A. , Leal, S. , Lafferty, M. , & Nahari, G. (2017). Insurance based lie detection: Enhancing the verifiability approach with a model statement component. Acta Psychologica, 174, 1–8. https://doi.org/10.1016/j.actpsy.2017.01.001 28088655 10.1016/j.actpsy.2017.01.001

[acp3407-bib-0016] Hauch, V. , Blandón‐Gitlin, I. , Masip, J. , & Sporer, S. L. (2015). Are computers effective lie detectors? A meta‐analysis of linguistic cues to deception. Personality and Social Psychology Review, 19(4), 307–342. 25387767 10.1177/1088868314556539

[acp3407-bib-0017] Honnibal, M. (2016). SpaCy (version 1.3.0). Retrieved from https://spacy.io/

[acp3407-bib-0018] Johnson, M. K. , Bush, J. G. , & Mitchell, K. J. (1998). Interpersonal reality monitoring: Judging the sources of other people's memories. Social Cognition, 16(2), 199–224.

[acp3407-bib-0019] Johnson, M. K. , & Raye, C. L. (1981). Reality monitoring. Psychological Review, 88(1), 67.

[acp3407-bib-0020] Jupe, L. M. , Leal, S. , Vrij, A. , & Nahari, G. (2017). Applying the verifiability approach in an international airport setting. Psychology, Crime & Law, (just‐accepted), 1–29.

[acp3407-bib-0021] Kleinberg, B. , Arntz, A. , & Verschuere, B. (in press). Detecting deceptive intentions: Possibilities for large‐scale applications In Docan‐Morgan T. (Ed.), The handbook of deceptive communication.

[acp3407-bib-0022] Kleinberg, B. , Mozes, M. , Arntz, A. , & Verschuere, B. (2017). Using named entities for computer‐automated verbal deception detection. Journal of Forensic Sciences.. https://doi.org/10.1111/1556-4029.13645 10.1111/1556-4029.13645 28940300

[acp3407-bib-0023] Kleinberg, B. , Nahari, G. , Arntz, A. , & Verschuere, B. (2017). An investigation on the detectability of deceptive intent about flying through verbal deception detection. Collabra: Psychology.. https://doi.org/10.1525/collabra.80

[acp3407-bib-0024] Kleinberg, B. , Nahari, G. , & Verschuere, B. (2016). Using the verifiability of details as a test of deception: A conceptual framework for the automation of the verifiability approach. In Proceedings of NAACL‐HLT (pp. 18–25). Retrieved from http://www.anthology.aclweb.org/W/W16/W16-0803.pdf

[acp3407-bib-0025] Köhnken, G. (2004). Statement validity analysis and the “detection of the truth”. The Detection of Deception in Forensic Contexts, 41–63.

[acp3407-bib-0026] Kuhn, M. (2017). caret: Classification and regression training (version R package version 6.0–76). Retrieved from https://cran.r-project.org/package=caret

[acp3407-bib-0027] Leal, S. , Vrij, A. , Warmelink, L. , Vernham, Z. , & Fisher, R. P. (2015). You cannot hide your telephone lies: Providing a model statement as an aid to detect deception in insurance telephone calls. Legal and Criminological Psychology, 20(1), 129–146.

[acp3407-bib-0028] Levine, T. R. , Blair, J. P. , & Carpenter, C. J. (2017). A critical look at meta‐analytic evidence for the cognitive approach to lie detection: A re‐examination of Vrij, Fisher, and Blank (2017). Legal and Criminological Psychology.. https://doi.org/10.1111/lcrp.12115

[acp3407-bib-0029] Mac Giolla, E. , Granhag, P. A. , & Liu‐Jönsson, M. (2013). Markers of good planning behavior as a cue for separating true and false intent: Good planning behavior and true and false intent. PsyCh Journal, 2(3), 183–189. https://doi.org/10.1002/pchj.36 26271363 10.1002/pchj.36

[acp3407-bib-0030] Masip, J. , Sporer, S. L. , Garrido, E. , & Herrero, C. (2005). The detection of deception with the reality monitoring approach: A review of the empirical evidence. Psychology, Crime & Law, 11(1), 99–122.

[acp3407-bib-0031] Meijer, E. H. , Verschuere, B. , & Merckelbach, H. (2017). Failing to tell friend from foe: A comment on Wijn et al. (2017). Legal and Criminological Psychology.. https://doi.org/10.1111/lcrp.12118

[acp3407-bib-0032] Mihalcea, R. , & Strapparava, C. (2009). The lie detector: Explorations in the automatic recognition of deceptive language. In Proceedings of the ACL‐IJCNLP 2009 Conference Short Papers (pp. 309–312). Association for Computational Linguistics Retrieved from http://dl.acm.org/citation.cfm?id=1667679

[acp3407-bib-0033] Murphy, K. P. (2012). Machine learning: A probabilistic perspective. MIT Press.

[acp3407-bib-0034] Nahari, G. (2016). When the long road is the shortcut: A comparison between two coding methods for content‐based lie‐detection tools. Psychology, Crime & Law, 22(10), 1000–1014.

[acp3407-bib-0035] Nahari, G. , Vrij, A. , & Fisher, R. P. (2014). Exploiting liars' verbal strategies by examining the verifiability of details. Legal and Criminological Psychology, 19(2), 227–239. https://doi.org/10.1111/j.2044-8333.2012.02069.x

[acp3407-bib-0036] Nothman, J. , Ringland, N. , Radford, W. , Murphy, T. , & Curran, J. R. (2013). Learning multilingual named entity recognition from Wikipedia. Artificial Intelligence, 194, 151–175.

[acp3407-bib-0037] Oberlader, V. A. , Naefgen, C. , Koppehele‐Goseel, J. , Quinten, L. , Banse, R. , & Schmidt, A. F. (2016). Validity of content‐based techniques to distinguish true and fabricated statements: A meta‐analysis. Law and Human Behavior, 40(4), 440–457. 27149290 10.1037/lhb0000193

[acp3407-bib-0038] Ormerod, T. C. , & Dando, C. J. (2015). Finding a needle in a haystack: Toward a psychologically informed method for aviation security screening. Journal of Experimental Psychology: General, 144(1), 76–84. https://doi.org/10.1037/xge0000030 25365531 10.1037/xge0000030

[acp3407-bib-0039] Ott, M. , Choi, Y. , Cardie, C. , & Hancock, J. T. (2011). Finding deceptive opinion spam by any stretch of the imagination. In Proceedings of the 49th Annual Meeting of the Association for Computational Linguistics: Human Language Technologies—Volume 1 (pp. 309–319). Association for Computational Linguistics Retrieved from http://dl.acm.org/citation.cfm?id=2002512

[acp3407-bib-0040] Pennebaker, J. W. , Boyd, R. L. , Jordan, K. , & Blackburn, K. (2015). The development and psychometric properties of LIWC2015. Retrieved from https://repositories.lib.utexas.edu/handle/2152/31333

[acp3407-bib-0041] Pérez‐Rosas, V. , & Mihalcea, R. (2014). Cross‐cultural deception detection. In ACL (2) (pp. 440–445). Retrieved from http://www.anthology.aclweb.org/P/P14/P14-2072.pdf

[acp3407-bib-0042] R Core Team (2016). R: A language and environment for statistical computing. Vienna, Austria: R Foundation for Statistical Computing Retrieved from https://www.r-project.org/

[acp3407-bib-0043] Robin, X. , Turck, N. , Hainard, A. , Tiberti, N. , Lisacek, F. , Sanchez, J.‐C. , & Müller, M. (2011). pROC: An open‐source package for R and S+ to analyze and compare ROC curves. BMC Bioinformatics, 12(1), 77. 21414208 10.1186/1471-2105-12-77 PMC3068975

[acp3407-bib-0044] Sooniste, T. , Granhag, P. A. , Knieps, M. , & Vrij, A. (2013). True and false intentions: Asking about the past to detect lies about the future. Psychology, Crime & Law, 19(8), 673–685. https://doi.org/10.1080/1068316X.2013.793333

[acp3407-bib-0045] Sooniste, T. , Granhag, P. A. , Strömwall, L. A. , & Vrij, A. (2015). Statements about true and false intentions: Using the cognitive interview to magnify the differences. Scandinavian Journal of Psychology, 56(4), 371–378. https://doi.org/10.1111/sjop.12216 25929812 10.1111/sjop.12216

[acp3407-bib-0046] Szpunar, K. K. (2010). Episodic future thought: An emerging concept. Perspectives on Psychological Science, 5(2), 142–162. https://doi.org/10.1177/1745691610362350 26162121 10.1177/1745691610362350

[acp3407-bib-0047] Venkatraman, E. S. (2000). A permutation test to compare receiver operating characteristic curves. Biometrics, 56(4), 1134–1138. 11129471 10.1111/j.0006-341x.2000.01134.x

[acp3407-bib-0048] Vrij, A. (2015). Verbal lie detection tools: Statement validity analysis, reality monitoring and scientific content analysis In Detecting deception: Current challenges and cognitive approaches (1st ed.) (pp. 3–35). Wiley Retrieved from https://books.google.nl/books?hl=en&lr=&id=4brlBQAAQBAJ&oi=fnd&pg=RA1-PA3&dq=Verbal+Lie+Detection+tools:+Statement+validity+analysis,+reality+monitoring+and+scientific+content+analysis&ots=4sFTBKx24S&sig=5lA5qnbszpbpaGcYokvw8n37ekw

[acp3407-bib-0049] Vrij, A. , Blank, H. , & Fisher, R. P. (2018). A re‐analysis that supports our main results: A reply to Levine et al. Legal and Criminological Psychology, 23(1), 20–23. https://doi.org/10.1111/lcrp.12121

[acp3407-bib-0050] Vrij, A. , Fisher, R. P. , & Blank, H. (2017). A cognitive approach to lie detection: A meta‐analysis. Legal and Criminological Psychology, 22(1), 1–21. https://doi.org/10.1111/lcrp.12088

[acp3407-bib-0051] Vrij, A. , & Granhag, P. A. (2012). Eliciting cues to deception and truth: What matters are the questions asked. Journal of Applied Research in Memory and Cognition, 1(2), 110–117. https://doi.org/10.1016/j.jarmac.2012.02.004

[acp3407-bib-0052] Vrij, A. , Granhag, P. A. , Mann, S. , & Leal, S. (2011). Lying about flying: The first experiment to detect false intent. Psychology, Crime & Law, 17(7), 611–620. https://doi.org/10.1080/10683160903418213

[acp3407-bib-0053] Vrij, A. , Granhag, P. A. , & Porter, S. (2010). Pitfalls and opportunities in nonverbal and verbal lie detection. Psychological Science in the Public Interest, 11(3), 89–121. https://doi.org/10.1177/1529100610390861 26168416 10.1177/1529100610390861

[acp3407-bib-0054] Vrij, A. , Hope, L. , & Fisher, R. P. (2014). Eliciting reliable information in investigative interviews. Policy Insights from the Behavioral and Brain Sciences, 1(1), 129–136.

[acp3407-bib-0055] Vrij, A. , Leal, S. , Mann, S. , Dalton, G. , Jo, E. , Shaboltas, A. , … Houston, K. (2017). Using the model statement to elicit information and cues to deceit in interpreter‐based interviews. Acta Psychologica, 177, 44–53. https://doi.org/10.1016/j.actpsy.2017.04.011 28477454 10.1016/j.actpsy.2017.04.011

[acp3407-bib-0056] Warmelink, L. , Vrij, A. , Mann, S. , & Granhag, P. A. (2013). Spatial and temporal details in intentions: A cue to detecting deception. Applied Cognitive Psychology, 27(1), 101–106. https://doi.org/10.1002/acp.2878

[acp3407-bib-0057] Warmelink, L. , Vrij, A. , Mann, S. , Jundi, S. , & Granhag, P. A. (2012). The effect of question expectedness and experience on lying about intentions. Acta Psychologica, 141(2), 178–183. 22964059 10.1016/j.actpsy.2012.07.011

[acp3407-bib-0058] Yarkoni, T. , & Westfall, J. (2017). Choosing prediction over explanation in psychology: Lessons from machine learning. Perspectives on Psychological Science, 12(6), 1100–1122. https://doi.org/10.1177/1745691617693393 28841086 10.1177/1745691617693393 PMC6603289

